# Esophageal cancer risk by type of alcohol drinking and smoking: a case-control study in Spain

**DOI:** 10.1186/1471-2407-8-221

**Published:** 2008-08-01

**Authors:** Jesus Vioque, Xavier Barber, Francisco Bolumar, Miquel Porta, Miguel Santibáñez, Manuela García de la Hera, Eduardo Moreno-Osset, the PANESOES Study Group

**Affiliations:** 1 Departamento Salud Pública, Universidad Miguel Hernández, Elche-Alicante, Spain; 2 CIBER de Epidemiología y Salud Pública, CIBERESP, Spain; 3 Departamento de Estadística, Centro de Investigación Operativa, Universidad Miguel Hernández de Elche-Alicante, Spain; 4 Facultad de Medicina, Universidad de Alcalá, Madrid, Spain; 5 Institut Municipal d'Investigació Mèdica, Universitat Autònoma de Barcelona, Spain; 6 Servicio de Medicina Digestiva, Hospital Universitario Dr. Peset, Universidad de Valencia, Valencia, Spain

## Abstract

**Background:**

The effect of tobacco smoking and alcohol drinking on esophageal cancer (EC) has never been explored in Spain where black tobacco and wine consumptions are quite prevalent. We estimated the independent effect of different alcoholic beverages and type of tobacco smoking on the risk of EC and its main histological cell type (squamous cell carcinoma) in a hospital-based case-control study in a Mediterranean area of Spain.

**Methods:**

We only included incident cases with histologically confirmed EC (n = 202). Controls were frequency-matched to cases by age, sex and province (n = 455). Information on risk factors was elicited by trained interviewers using structured questionnaires. Multiple logistic regression was used to estimate adjusted odds ratios and 95% confidence intervals (CI).

**Results:**

Alcohol drinking and tobacco smoking were strong and independent risk factors for esophageal cancer. Alcohol was a potent risk factor with a clear dose-response relationship, particularly for esophageal squamous-cell cancer. Compared to never-drinkers, the risk for heaviest drinkers (≥ 75 g/day of pure ethanol) was 7.65 (95%CI, 3.16–18.49); and compared with never-smokers, the risk for heaviest smokers (≥ 30 cigarettes/day) was 5.07 (95%CI, 2.06–12.47). A low consumption of only wine and/or beer (1–24 g/d) did not increase the risk whereas a strong positive trend was observed for all types of alcoholic beverages that included any combination of hard liquors with beer and/or wine (p-trend<0.00001). A significant increase in EC risk was only observed for black-tobacco smoking (2.5-fold increase), not for blond tobacco. The effects for alcohol drinking were much stronger when the analysis was limited to the esophageal squamous cell carcinoma (n = 160), whereas a lack of effect for adenocarcinoma was evidenced. Smoking cessation showed a beneficial effect within ten years whereas drinking cessation did not.

**Conclusion:**

Our study shows that the risk of EC, and particularly the squamous cell type, is strongly associated with alcohol drinking. The consumption of any combination of hard liquors seems to be harmful whereas a low consumption of only wine may not. This may relates to the presence of certain antioxidant compounds found in wine but practically lacking in liquors. Tobacco smoking is also a clear risk factor, black more than blond.

## Background

Most epidemiological studies have identified tobacco smoking and alcohol drinking as the main risk factors for esophageal squamous-cell carcinoma or unspecified esophageal cancer [1-3], usually with a monotonic and strong dose-response relationship [4]. The evidence that high alcohol consumption increases the risk of cancer of the esophagus is quite convincing at present; most of it suggests that it is the amount of alcohol consumed, rather than the particular drink that determines the risk. However, a prospective cohort study in Denmark observed that low wine consumption, compared to beer and hard liquor, did not increase risk [5]. Two population-based case-control studies also presented data supporting this observation [6,7]. In an ecological study in Spain, trends of per capita consumption of beer, spirits, and total alcohol consumption were positively correlated with oesophageal cancer mortality in men whereas wine consumption showed no relationship with oesophageal cancer mortality either in men or women[8]. As the consumption of all types of alcoholic drinks is quite prevalent in Spain, it is relevant to assess the risk of esophageal cancer in relation to them.

Tobacco smoking has also a clear role in the aetiology of esophageal cancer [1,2]. Despite the high prevalence of consumption, particularly of black tobacco, an association between the type of tobacco smoking and esophageal cancer has never been explored by epidemiological studies in Spain, only in South America or France[4,9].

In addition to smoking and alcohol, other factors such as a low socioeconomic status, an infrequent consumption of fruits and vegetables and, in some areas of Asia, the betel nut chewing, have also been related to esophageal cancer [10-13]; these factors account for a high proportion of cases, particularly for the esophageal squamous cell type, the most frequent histological type [11,12,14].

We conducted a case-control study in Valencia and Alicante, Spain, to estimate the independent effects of different alcoholic beverages (beer, wine and spirits) and type of tobacco smoking (black and blond) on the risk of esophageal cancer and its main histological cell type (squamous cell carcinoma).

## Methods

### Design

This research was part of the PANESOES project, a prospective hospital-based case-control study designed to explore the influence of major lifestyles and diet on the risk of three gastrointestinal cancers, pancreas, oesophagus and stomach. The PANESOES Study aimed to recruit approximately 200 cases for oesophagus, 200 for pancreas cancer, 400 cases for stomach cancer, and 400–450 controls. This sample size was planned to estimate as statistically significant (p < 0.05) a RR over 1.5 for stomach cancer and high prevalent exposures such as tobacco smoking and alcohol drinking, and a RR over 1.8 for oesophagus and pancreas cancer.

Eligible subjects were Spanish-speaking men and women 30–80 years old, and hospitalized between January 1995 and March 1999 in any of nine participant hospitals in the provinces of Alicante and Valencia. These nine hospitals are among the ten main hospitals from the Health Care Service in Valencia and Alicante that were invited to participate (only one hospital in Valencia declined participation). The access to the Health Care System in Spain is free and universal; thus, our case series may be considered representative as the participant hospitals accounted for approximately 90% of cases in both provinces.

All subjects were informed of the study objectives and gave their informed consent before the interview. Research protocols were approved by the local ethics and/or research committees of the participating Hospitals and the University.

### Subjects

Cases were patients newly diagnosed with a primary invasive cancer of the oesophagus. Pathology reports were obtained for all case patients initially diagnosed as esophageal cancer, and final inclusion in the case series was based on histological confirmation. A total of 211 cases were initially identified as eligible for participation; two cases refused interview (0.9%), and seven cases were not finally confirmed (3.3%). The final analysis was thus based on 202 cases. The pathological diagnosis was confirmed as squamous cell carcinoma in 160 cases (79.2%) and adenocarcinoma in 42 cases (20.8%).

Control subjects were selected from the same hospitals from which cases were identified and during the same period. Controls were frequency matched to the expected distribution of case subjects of the whole PANESOES study (i.e. cases of oesophagus, stomach and pancreas, and controls) by three age groups (<60 years, 60–69 and 70–80 years), sex, and province (Alicante and Valencia). A wide inclusion criterion was used to select controls from diseases not related a priori to the main exposures of interest (tobacco, alcohol and diet). The overall participation rate of the 457 eligible controls was 99.6%, leaving 455 controls subjects with completed interviews. The distribution of the main diagnoses for the control group was, in decreasing order: hernia (34.0%), degenerative osteoarthritis/arthritis (21.3%), fractures/injuries/orthopaedic processes (18.9%), appendicitis (6.4%) and other, less prevalent, conditions (19.3%).

### Exposure data

Face-to-face interviews were conducted in-hospital for all participants by trained interviewers, using a structured questionnaire. Interviews were administered directly to the study subjects, rather than to the next of kin, for 89.2% of the target case subjects and for 96% of the target control subjects. The average time required to complete the questionnaire was 40 minutes. While interviewers could not be blinded to the case/control status, they were unaware of the main study hypothesis and were trained to administer strictly the structured questionnaires in an equal manner to case and controls.

Information was collected on demographic characteristics, tobacco and alcohol use, medical history and other lifestyle factors. The interview elicited details on usual tobacco use, including the product/tobacco type (black or blond cigarettes, cigars, pipes) as well as intensity of use, the age at which the habit started and stopped, the total duration of use excluding the years stopped, the years since last use for each type of product, and the level of inhalation when smoking (partially or totally). A never smoker was defined as someone having smoked fewer than 100 cigarettes ever or less than one cigarette per day for one year. A former smoker was defined as someone having stopped smoking 1 or more years before the interview. We computed the average number of cigarettes smoked per day including type of tobacco smoked, lifetime number of cigarettes smoked and pack-years of smoking (number of 20-cigarette packs per day multiplied by number of years smoking). Only 2 cases and 8 controls simultaneously smoked both blond and black tobacco, and they were considered as mainly blond tobacco smokers.

Alcohol consumption patterns were assessed through inquiries into the usual intake for each type of beverage separately, i.e. beer, wine, or liquor. Since the intake of only wine and/or beer intake was uncommon among cases, these two alcoholic beverages were combined in the analysis. Since only 8 participants consumed liquors but not beer or wine, we estimated a category for the combined consumption of liquors with any combination of beer and/or wine (All types of beverages). The average relative contributions of beer, wine and liquor consumption in this category were 30%, 50% and 20% respectively. The three types of alcohol were also combined to give an overall estimate of the alcohol consumption. A never drinker was defined as having consumed less than one drink per month. One drink was defined as 200 cc of beer, 125 cc of wine, or 50 cc of hard liquor. The content of pure alcohol was calculated according to the following concentrations specific for Spain: 5% for beer; 12 percent for wine; and 40 percent for hard liquors. The resulting values were converted to grams multiplying 1 ml of pure ethanol per 789 mg [15]. The average in grams of pure ethanol consumed per day, type of alcoholic beverage, and life time duration of the habit, the age at starting and stopping the habit, and then the years since quitting drinking were also estimated. A former drinker was defined as having stopped drinking 1 or more years before the interview.

The fruit and vegetable intake was assessed by a food frequency questionnaire (FFQ). For this study, we adapted and validated a FFQ of 93 food items similar to the Harvard questionnaire in order to assess the diet five years before the interview in the hospital [16-18]. Participants were asked to report their average consumption of 12 vegetables and 10 fruit items. The average daily intakes for each fruit and vegetable were summed to compute the total fruit and vegetable intake in grams, and they were adjusted for energy intake using the residual method [19]. We further computed tertiles of energy-adjusted intake of fruit and vegetable using the distribution of cases and controls in the whole PANESOES study (cases of oesophagus, stomach and pancreas, and controls).

### Statistical Analysis

We used unconditional logistic regression to estimate odds ratios (OR), as an estimate of relative risk, and corresponding 95% confidence intervals (CIs) [20]. All regression models included as covariates the three frequency-matched factors, sex (men/women), age (<60 years, 60–69 years, 70–80 years) and hospital origin (Valencia/Alicante) entered as indicator variables; the educational level (<primary, primary completed, and ≥ secondary school entered as indicator variable); and the different variables of alcohol intake and tobacco smoking also using indicator variables. In addition, we adjusted for a potential confounding effect of fruit and vegetable intake (in tertiles and entered as indicator variables), and energy intake.

Tests for trend in the ORs across exposure strata were calculated for ordinal variables by using logistic models that included categorical terms as continuous variables in a model with all the potential confounders and, where appropriate, omitting the never or former users/exposed. For trend-tests, we used the likelihood ratio test statistic with one degree of freedom. Although the study sample size did not allow us to estimate tests of interactions, we performed exploratory analyses for the effects of alcohol and tobacco by strata of never-ever smoking status and never-ever drinking status, respectively. All analyses were performed with STATA-8 [21]. Statistical significance was set at 0.05. All reported *P *values are from two-sided tests.

## Results

Table 1 shows the distribution of cases and controls according to demographic characteristics and main exposure variables. The distribution of the frequency-matched variables age, sex and province were comparable between the control series and the overall case series of the PANESOES study (i.e., cancers of oesophagus, stomach and pancreas, data not shown). The educational level was comparable between cases and controls. Alcohol intake, tobacco smoking and a low intake of fruit and vegetable were more prevalent among cases than controls.

**Table 1:** Distribution of socio-demographic characteristics and other exposure variables among case and control subjects

**Variables**	**Cases^a^**	**%**	**Controls**	**%**
**Sex**				
Men	187	92.61	285	62.64
Women	15	7.39	170	27.78
				
**Age**				
<60 years old	86	42.36	149	32.75
60–69 years old	79	39.41	167	36.70
≥ 70 years old	37	18.23	139	30.55
				
**Province**				
Alicante	45	22.66	139	30.55
Valencia	157	77.34	316	69.45
				
**Educational level**				
<Primary	114	56.65	246	54.07
Primary	67	33.00	172	37.80
≥ Secondary	21	10.34	37	8.13
				
**Alcohol intake**				
Never	16	7.88	171	37.36
Ever	186	92.12	284	62.64
				
**Tobacco smoking**				
Never smoker	23	11.39	218	47.91
Ever smoker	179	88.61	237	52.09
				
**Fruit and Vegetable daily intake (in tertile)**				
< 166 g/d	121	59.90	104	22.86
166–255 g/d	43	21.29	141	30.99
>255 g/d	38	18.81	210	46.15

^a ^All cases were histologically confirmed.

Table 2 shows risk estimates according to various patterns of alcohol intake. We identified moderate to strong effects for all measures of amount and duration (years of alcohol drinking); risk was particularly strong for all types of beverages. Former drinkers and current drinkers experienced, respectively, a 4.3 and 2.1-fold increase of risk over never drinkers. An increasing risk of esophageal cancer was observed according to the daily amount of pure ethanol consumption (p-trend < 0.00001); risk was very high and significant among subjects consuming ≥ 75 g/d (OR = 7.65). Concerning the beverage type, drinkers of all types of beverages showed the highest risk (OR = 4.39) and ever drinkers of only beer also showed a significant effect (3.07); however, no significant effects were observed for drinkers of only wine or wine and/or beer. When the daily amount by type of beverage was considered, no significantly increased risks were observed for wine-beer combined consumption. For all types of alcoholic beverages combined, a non-significant risk was observed for a daily consumption of 1–24 g/day (OR = 1.53), whereas risk increased sharply to 3.9 and 10.6-fold for the upper categories (p-trend<0.0001).

**Table 2:** Adjusted^a ^odds ratios (OR) for esophageal cancer, and esophageal squamous-cell carcinoma, according to alcohol consumption

	**No. of controls**	**All esophageal cancer cases**			**Esophageal squamous-cell carcinoma cases**		
		
		**Cases**	**OR^a^**	**95% CI**	**Cases**	**OR^a^**	**95% CI**
**Alcohol Drinking Status**							
Never<	171	16	1.00		6	1.00	
Ever	284	186	2.41	(1.24 – 4.71)	154	5.34	(2.05 – 13.91)
Never	171	16	1.00		6	1.00	
Former drinker	49	38	4.28	(1.92 – 9.56)	31	11.03	(3.73 – 32.62)
Current drinker	235	148	2.06	(1.04 – 4.08)	123	4.48	(1.69 – 11.83)
**Average of pure ethanol (g/day)**							
Never	171	16	1.00		6	1.00	
Former drinker	49	38	5.40	(2.43 – 12.00)	31	16.03	(5.34 – 48.07)
1–24 g/d	147	27	1.16	(0.54 – 2.49)	12	1.71	(0.56 – 5.20)
25–74 g/d	62	45	2.89	(1.29 – 6.48)	38	8.02	(2.64 – 24.40)
≥ 75 g/d	26	75	7.65	(3.16 – 18.49)	72	23.20	(7.19 – 74.90)
***p-value for linear trend***				***<0.00001***			***<0.00001***
**Type of drink (g/day)**							
Never	171	16	1.00		6	1.00	
Only wine	65	12	1.20	(0.49 – 2.91)	7	1.92	(0.56 – 6.64)
Only beer	26	8	3.07	(1.06 – 8.90)	6	7.98	(2.13 – 29.92)
Only wine and/or beer	67	15	1.44	(0.59 – 3.47)	9	2.49	(0.73 – 88.46)
All types of beverages	126	151	4.39	(2.08 – 9.22)	132	12.15	(4.17 – 34.56)
Never	171	16	1.00		6	1.00	
Former drinker	49	38	5.50	(2.47 – 12.27)	31	16.74	(5.53 – 50.67)
Wine and/or Beer 1–24 g/d	107	14	1.04	(0.45 – 2.41)	6	1.48	(0.43 – 5.05)
Wine and/or Beer ≥ 25 g/d	30	13	2.04	(0.76 – 5.46)	11	5.48	(1.52 – 19.71)
All types 1–24 g/d	40	13	1.53	(0.58 – 4.01)	6	2.47	(0.62 – 9.86)
All types ≥ 25 g/d	58	108	5.76	(2.59 – 12.83)	100	17.22	(5.68 – 52.21)
All types 25–74 g/d	40	39	3.88	(1.64 – 9.15)	32	10.47	(3.26 – 33.64)
All types ≥ 75 g/d	18	69	10.62	(4.14 – 27.24)	68	35.03	(10.28 – 119.31)
***p-value for linear trend***				***<0.0001***			***<0.0001***
**Years of alcohol drinking**							
0	171	16	1.00		6	1.00	
1–19	16	15	4.06	(1.37 – 12.00)	11	8.21	(2.08 – 32.32)
20–39	121	92	2.96	(1.42 – 6.14)	76	6.28	(2.27 – 17.35)
≥ 40	129	71	1.71	(0.80 – 3.61)	62	3.99	(1.41 – 11.24)
***p-value for linear trend***				***0.316***			***0.036***
**Age at starting**							
<18 yr	51	43	1.00		39	1.00	
18–20 yr	88	50	1.94	(0.88 – 4.27)	40	0.63	(0.29 – 1.37)
21–29 yr	59	34	1.04	(0.53 – 2.02)	31	0.83	(0.34 – 2.02)
≥ 30 yr	72	52	1.71	(0.86 – 3.40)	40	0.91	(0.36 – 2.29)
**Years since quitting**							
Current Drinker	235	148	1.00		123	1.00	
<5 yr	12	16	3.60	(1.34 – 9.69)	14	5.89	(2.01 – 17.25)
≥ 5 yr	37	22	1.71	(0.86 – 3.41)	17	1.70	(0.79 – 3.66)

^a ^Adjusted for sex, age, educational level, province and tobacco smoking (never, past, <20, 20–49 and ≥ 50 pack-years), the energy-adjusted intake of fruit and vegetable in tertiles, and energy intake.

Regarding duration of drinking, statistically significant increased risks were observed for up to 40 years of drinking, after which no significant increase was observed (Table 2). Age at start drinking was not significantly associated to a higher risk of esophageal cancer. Drinking cessation in the last 5 years was associated with a strong risk excess compared with persistent drinkers (OR = 3.60, 95% CI 1.34–9.69); the risk decreased thereafter although still remained higher than in current drinkers.

Table 2 also shows the estimated ORs according to various patterns of alcohol intake specifically for the esophageal squamous-cell carcinoma histological type (n = 160 cases). Most effects were approximately 3 times higher than risk estimates for the whole case series (n = 202 cases). The effects of all types of alcoholic beverages, were in general 2 to 4-fold higher than those observed for drinkers of only wine and/or beer. The highest risk of esophageal cancer was observed among cases with an average daily intake of pure ethanol ≥ 75 g/d (OR = 23.20, 95% CI: 7.19–74.90), and particularly for those consuming ≥ 75 g/d of all types of alcoholic beverages (OR = 35.03, 95% CI: 10.28–119.31). No significant effects were observed for only wine or beer drinkers 1–24 g/d.

Table 3 shows adjusted risk estimates according to various patterns of tobacco smoking. The risk of esophageal cancer was more than doubled among ever smokers. Current smokers presented a 2.6-fold increase of risk with respect to never smokers. The adjusted ORs increased with the number of cigarettes smoked per day up to 5 among cases smoking ≥ 30 cigarettes/day (p-trend = 0.002), and with increasing years of smoking (p-trend = 0.030). Pack-years also showed a significant dose-response (p-trend = 0.005). No association was found with age at which subjects started smoking. After smoking cessation there was a reduction of risk of 35% for ten or more years and 45% for less than ten years that overall was statistically significant (LRS, p-value = 0.042). A statistically significant increased risk was observed for black tobacco but not for blond tobacco. Smokers who totally inhaled the tobacco experienced a statistically significant higher risk than non- or partially inhaling smokers. The risks for esophageal squamous-cell carcinoma were very similar to those observed for the whole case series.

**Table 3:** Adjusted^a ^odds ratios (OR) for esophageal cancer, and esophageal squamous-cell carcinoma, according to tobacco smoking

	**No. of controls**	**All esophageal cancer cases**			**Esophageal squamous-cell carcinoma cases**		
		
		**Cases**	**OR^a^**	**95% CI**	**Cases**	**OR^a^**	**95% CI**
**Smoking Status**							
Never	218	23	1.00		15	1.00	
Ever	237	179	2.12	(1.06 – 4.23)	145	1.70	(0.72 – 4.03)
Never	218	23	1.00		15	1.00	
Former-Smoker	117	55	1.61	(0.76 – 3.40)	39	1.08	(0.43 – 2.73)
Current Smoker	120	124	2.58	(1.26 – 5.28)	106	2.28	(0.95 – 5.51)
**Average No. of cigarettes/day**							
Never	218	23	1.00		15	1.00	
Former smoker	117	55	1.68	(0.79 – 3.57)	39	1.16	(0.45 – 2.99)
<15	27	11	1.70	(0.63 – 4.58)	8	1.35	(0.41 – 4.48)
15–29	58	58	2.45	(1.11 – 5.44)	47	2.05	(0.78 – 5.42)
≥ 30	23	51	5.07	(2.06 – 12.47)	48	5.82	(1.96 – 17.23)
Pipe/Cigars	12	4	1.58	(0.41 – 6.21)	3	1.49	(0.30 – 7.46)
***p-value for linear trend***				***0.002***			***0.006***
**Years of cigarette smoking**							
Never	218	23	1.00		15	1.00	
<20	39	14	1.78	(0.69 – 4.59)	4	0.54	(0.12 – 2.11)
20–29	48	32	1.94	(0.81 – 4.63)	26	1.62	(0.57 – 4.65)
≥ 30	150	134	2.25	(1.10 – 4.58)	115	2.03	(0.84 – 4.92)
***p-value for linear trend***				***0.030***			***0.029***
**Pack-years^b^**							
Never smoker (0 p-y)	218	23	1.00		15	1.00	
Past	117	55	1.67	(0.79 – 3.55)	39	1.15	(0.45 – 2.95)
<20	38	11	1.30	(0.49 – 3.47)	6	0.69	(0.19 – 2.51)
20–49	55	59	2.81	(1.27 – 6.19)	48	2.49	(0.96 – 6.51)
≥ 50	27	54	3.79	(1.60 – 8.95)	52	3.93	(1.40 – 10.99)
***p-value for linear trend***				***0.005***			***0.007***
**Age at starting**							
<15	40	41	1.00		35	1.00	
15–17	78	61	1.11	(0.56 – 2.21)	49	1.45	(0.65 – 3.23)
≥ 18	116	70	0.91	(0.47 – 1.74)	56	1.21	(0.57 – 2.58)
**Years since quitting**							
Current Smoker	120	124	1.00		106	1.00	
<10	50	26	0.55	(0.29 – 1.06)	20	0.44	(0.20 – 0.96)
≥ 10	67	29	0.65	(0.35 – 1.21)	19	0.49	(0.23 – 1.06)
**Type of tobacco**							
Never	218	23	1.00		15	1.00	
Blond	71	31	1.19	(0.50 – 2.82)	24	0.80	(0.28 – 2.30)
Black	150	144	2.50	(1.23 – 5.09)	118	2.05	(0.85 – 4.97)
**Inhalation of smoke**							
Never	218	24	1.00		15	1.00	
Partially	164	93	1.50	(0.74 – 3.07)	77	1.32	(0.54 – 3.25)
Totally	72	85	2.88	(1.37 – 6.06)	67	2.53	(1.00 – 6.46)

^a ^Adjusted for sex, age, educational level, province, and alcohol drinking (never, past, 1–24 g/d, 25–74 g/d, ≥ 75 g/d), the energy-adjusted intake of fruits and vegetables in tertiles, and energy intake

^b ^Pack-years = the number of packs of 20 cigarettes per day multiplied by the number of years smoking.

## Discussion

Our results confirm that alcohol drinking and tobacco smoking are both strong and independent risk factors for esophageal cancer in Spain. We found that heavy drinkers had higher increased risk than heavy smokers, particularly for the esophageal squamous-cell carcinoma. These results are consistent with most of the epidemiological studies carried out in Western countries and some areas of Asia [4,11,12,22-25]. Other studies have found however, chewing or tobacco smoking to be a similar or even a stronger risk factor than alcohol drinking [13,26-28] although some of these studies did not include subjects with a high consumption of alcohol [13,26].

Regarding alcohol drinking, we found that the intensity (i.e. the average daily alcohol intake in grams of pure ethanol) was a more relevant predictor of risk than the duration of the habit. The age at which subjects started drinking was not associated with risk and the cessation of drinking was not associated with any beneficial effect even ≥ 5y after cessation. The consequences of drinking cessation has been studied less frequently than smoking cessation and results are more controversial, probably because the number of people who quit drinking is lower than those who quit smoking. A beneficial effect has been found in some studies particularly 10 years after giving up drinking [25,29] although some studies have shown a rapid decline in risk after cessation of drinking [4,30,31]. On the contrary, other studies have shown either a non beneficial effect [23] or a higher risk among former drinkers [31,32]. In our study we observed a statistically significant increased risk among former drinkers who stopped drinking <5y. Unfortunately, we had an insufficient number of former drinkers to explore the drinking cessation after 10 years in more detail. Although we considered as former drinkers those who reported quitting at least one year before the interview, it is possible that some of the patients were in fact heavy drinkers quitting the habit because of their disease; alternatively, it may also be possible that some heavy drinker cases misreported their habit, declaring no consumption when in fact they were still drinking and consequently exhibited a higher risk than never drinkers.

We were also interested in exploring the effect of the alcohol type since the consumption of wine, beer and spirits is quite common in Spain [33] and thus, it may have a great interest for public health. Most of the studies have supported the hypothesis that the amount of alcohol consumed is the determinant of the risk rather than the particular drink, however, a prospective cohort study in Denmark showed that a moderate intake of wine, compared to beer and hard liquor, did not increase the risk [5]. Previously, two population-based case-control studies in the United States did not find an association between wine drinking and the risk of esophageal cancer [34,35], and later, two other studies showed data supporting a lack of effect for wine drinking as well [6,7]. Conversely, in other studies where wine was by far the most common alcoholic beverage, wine drinkers showed higher risk than those of other beverages [23,36]. In our study, the consumption of all types of beverages that include hard liquors was a much stronger risk factor than wine and/or beer. We observed a statistically significant increased risk of EC among only beer drinkers but not among only wine or wine/beer drinkers. When the daily amount by type of beverage was considered, no increased risks were observed for a low-moderate wine-beer consumption (1–24 d/day), and a 2-fold increase risk among drinkers of ≥ 25g/d although it was not statistically significant. However, we observed a 5.76-fold increase risk among drinkers of ≥ 25 g/d of all types of beverages (risk for the combined categories of 25–74 g/d and ≥ 75 g/d of all types of beverages, table 2). A low-moderate wine-beer consumption of 1–24 g/d did not increase significantly the risk of esophageal squamous-cell carcinoma either. If the lack of effect of wine and/or beer is real, there may be some protective ingredients in wine such as resveratrol [37] and other antioxidants that may cause such a protective effect [38].

It is not clear the exact mechanisms by which alcoholic beverages induce esophageal cancer risk. An ingredient common to all beverages is ethanol although it is possible that other components or contaminants such as N-nitrosamines and urethane with carcinogenic properties may increase cancer risk. It has been shown in practically all the studies that the risks are greatest for drinkers of hard liquors which is consistent with evidence that the concentration of ethanol plays an important role in alcohol-related tumours of the upper aero-digestive tract [13,39,40]. Although ethanol has not been shown as carcinogenic in laboratory animals, it may act through its major metabolite, acetaldehyde, a carcinogen in animal models [41,42]. Thus, it has been suggested that in addition to a systemic effect, ethanol can be converted to acetaldehyde in saliva and exert a promoting effect by either solubilising tobacco-specific carcinogens or enhancing their penetration into the esophageal mucosa, by nutritional deficiencies associated with heavy drinking or by other mechanisms (e.g., direct toxic or oxidative effect on the epithelial mucosa) [41,43]. In order to explain the differential effect of alcoholic beverages it should be considered that certain compounds such as N-nitrosamines are found in liquors and to lesser extent in beer, and urethane, a potential carcinogen in experimental studies, may be found in liquor but not in beer and wine. In addition, while most of the antioxidant compounds found in wine and to some extent in beer, are almost completely lacking in spirits [38].

It has been also suggested that part of the effect observed for alcohol and/or tobacco smoking could be due to other factors or different lifestyles, such as a low fruit and vegetable intake [10]. However, our data were adjusted for the intake of fruit and vegetables showing that alcohol and tobacco were strong risk factors although the risk estimates were much higher when we did not adjust for fruits and vegetables intake (data not shown). We also observed that alcohol drinking was a much stronger risk factor for squamous-cell carcinoma than for the whole control series. This finding would indirectly support the hypothesis that the effect of alcohol for adenocarcinoma may be much weaker, if any. In fact, when we estimated the risk of adenocarcinoma for wine-beer drinkers and all types of alcohol beverages with respect to never drinkers, we did not find any increase of risk (OR = 0.83 and OR = 0.97, respectively); and for former and current drinker *vs *never drinkers, we did not find any significant risk either (OR = 1.83 and OR 0.74, respectively). Although some studies have found slight excesses of adenocarcinoma among drinkers [34,35,44], most of the previous case-control studies between alcohol drinking and the risk of oesophageal adenocarcinoma have generally found no association [6,7,45-47]. Unfortunately, the number of adenocarcinoma cases in our study was too small (n = 42) to allow us a separate analysis in great detail. The reasons for the differential effect of alcohol drinking on oesophageal squamous cell-carcinoma and adenocarcinoma should be further examined.

Concerning tobacco smoking, the effect estimates were lower than the observed for alcohol intake although dose-response trends were still evident for the amount and the duration of smoking. Although the effect of tobacco smoking has been considered by practically all studies on esophageal cancer only a few have explored the effects by type of tobacco black/blond. The increased risk of black tobacco compared to blond tobacco is consistent with ecological studies showing a relatively high incidence of cancers of upper aero-digestive tract in southern Europe and Latin America, where this kind of tobacco is mainly consumed [48]. Our results are consistent with other studies carried out in high-risk areas of South America [4,49]. We observed a statistically significant effect for black tobacco but not for blond tobacco although the lack of effect for low-to-moderate blond tobacco smoking was based on a small number of cases (n = 31). It has been suggested that the higher concentrations of some carcinogenic compounds such as N-nitrosamines found in smoke of black tobacco could be a mechanism involved [43].

Unlike our findings for alcohol cessation, we observed a beneficial effect of cessation of smoking. Similarly to others [4,6], we found that the risk of esophageal cancer declined within a decade of smoking cessation which may suggest that smoking could act during later stages as a promoter in the development of esophageal cancer, predominantly for the esophageal squamous-cell carcinoma.

In order to explore the joint effects of alcohol and tobacco, unfortunately we could not perform interaction tests because of the small numbers. There was evidence that the effect of both exposures were nearly multiplicative. If we accept a multiplicative effect, we could assume that the risk of being simultaneously a heavy drinker (≥ 75 g/d) and heavy smoker (>50 p/y) could be as high as 40 times that of abstainers, or even 100 times when referring to esophageal squamous-cell carcinoma.

As in other case-control studies, this study may present limitations. The sample size was small, and we could not explore interaction between tobacco and alcohol in more detail but the effects of each exposure became evident even among those non-exposed to the other. Another limitation may relate to the use of hospital controls. It is known that controls in hospital-based studies may be heavier smokers and heavier drinkers compared with general population; but we used a wide criterion to select controls from diagnosis a priori not related to the main risk factors of our study. In fact, the prevalence of drinking or smoking among control subjects was similar to that observed in the adult general population of the same sex and age composition of Alicante and Valencia, and thus it could be considered as representative of that population[33]. The presence of other types of bias were minimized by selecting controls from the same hospital as the cases, using the same interviews and procedures in both cases and controls to avoid any differential misclassification. The strength of the associations, the existence of a dose-response, the reduction of effect after cessation, the control for other potential confounder such as education and the intake of fruits and vegetables, and the consistency with other studies would support that the study results are real, and that alcohol drinking and tobacco smoking are likely to be causally related to esophageal cancer.

## Conclusion

In conclusion, this case-control study shows that the risk of esophagus cancer, and particularly the squamous cell type, is strongly associated with alcohol drinking. The consumption of any combination of hard liquors seems to be harmful whereas a low consumption of only wine may not. Tobacco smoking is also a strong risk factor, black more than blond. Smoking cessation was shown a beneficial effect within ten years whereas drinking cessation was not. A possible differential effect of alcohol drinking on oesophageal squamous cell-carcinoma (harmful) and adenocarcinoma (no effect) should be further examined.

## Competing interests

The authors declare that they have no competing interests

## Authors' contributions

JV, Principal Investigator, conceived, designed and coordinated the study, performed the statistical analysis and drafted the manuscript; XB, performed the statistical analysis; FB, MP, MS and MGdlH made substantial contributions to the interpretation of data and drafting the manuscript; EM-O, made substantial contributions to acquisition of data, verifying the diagnoses for case series and drafting the manuscript. All the authors read and approved the final manuscript.

## Pre-publication history

The pre-publication history for this paper can be accessed here:


